# *PHIP*-associated Chung-Jansen syndrome: Report of 23 new individuals

**DOI:** 10.3389/fcell.2022.1020609

**Published:** 2023-01-16

**Authors:** Antje Kampmeier, Elsa Leitão, Ilaria Parenti, Jasmin Beygo, Christel Depienne, Nuria C Bramswig, Tzung-Chien Hsieh, Alexandra Afenjar, Stefanie Beck-Wödl, Ute Grasshoff, Tobias B Haack, Emilia K Bijlsma, Claudia Ruivenkamp, Eva Lausberg, Miriam Elbracht, Maria K Haanpää, Hannele Koillinen, Uwe Heinrich, Imma Rost, Rami Abou Jamra, Denny Popp, Margarete Koch-Hogrebe, Kevin Rostasy, Vanesa López-González, María José Sanchez-Soler, Catarina Macedo, Ariane Schmetz, Carmen Steinborn, Sabine Weidensee, Hellen Lesmann, Felix Marbach, Pilar Caro, Christian P. Schaaf, Peter Krawitz, Dagmar Wieczorek, Frank J Kaiser, Alma Kuechler

**Affiliations:** ^1^ Institut für Humangenetik, Universitätsmedizin Essen, Universität Duisburg-Essen, Essen, Germany; ^2^ Institut für Genomische Statistik und Bioinformatik, Universitätsklinikum Bonn, Rheinische Friedrich-Wilhelms-Universität Bonn, Bonn, Germany; ^3^ Département de génétique et embryologie médicale, Centre de Référence Malformations et maladies congénitales du cervelet et déficiences intellectuelles de causes rares, Hôpital Trousseau, APHP Sorbonne Université, Paris, France; ^4^ Institute of Medical Genetics and Applied Genomics, University of Tübingen, Tübingen, Germany; ^5^ Department of Clinical Genetics, Leiden University Medical Center, Leiden, Netherlands; ^6^ Institut für Humangenetik und Genommedizin, Uniklinik RWTH Aachen, Aachen, Germany; ^7^ Clinical Genetics Unit, Turku University Hospital, Turku, Finland; ^8^ Department of Genomics, Turku University Hospital, Turku, Finland; ^9^ Institute of Biomedicine, University of Turku, Turku, Finland; ^10^ Zentrum für Humangenetik und Laboratoriumsdiagnostik Dr. Klein Dr. Rost und Kollegen, Martinsried, Germany; ^11^ Institute of Human Genetics, University of Leipzig Medical Center, Leipzig, Germany; ^12^ Vestische Kinder- und Jugendklinik Datteln, Abteilung für Neuropädiatrie, Datteln, Germany; ^13^ Sección Genética Médica, Servicio de Pediatría, Hospital Clínico Universitario Virgen de la Arrixaca, Murcia, Spain; ^14^ Sección de Genética Médica, Servicio de Pediatría, Hospital Clínico Universitario Virgen de la Arrixaca, IMIB-Arrixaca, CIBERER, Murcia, Spain; ^15^ Serviço de Genética, Departamento de Pediatria, Hospital de Santa Maria, Centro Hospitalar e Universitário Lisboa Norte, Centro Académico de Medicina de Lisboa, Lisboa, Portugal; ^16^ Institute of Human Genetics, Medical Faculty, University Hospital Düsseldorf, Heinrich Heine University Düsseldorf, Düsseldorf, Germany; ^17^ MVZ Mitteldeutscher Praxisverbund Humangenetik, Dresden, Germany; ^18^ MVZ Mitteldeutscher Praxisverbund Humangenetik, Erfurt, Germany; ^19^ Institut für Humangenetik, Universitätsklinikum Bonn, Universität Bonn, Bonn, Germany; ^20^ Institut für Humangenetik, Universitätsklinikum Heidelberg, Universität Heidelberg, Heidelberg, Germany; ^21^ Center for Rare Diseases, Medical Faculty, University Hospital Düsseldorf, Heinrich Heine University Düsseldorf, Düsseldorf, Germany; ^22^ Essener Zentrum für Seltene Erkrankungen (EZSE), Universitätsmedizin Essen, Essen, Germany

**Keywords:** Chung-Jansen syndrome, CHUJANS, PHIP, DIDOD syndrome, ID, DD, obesity, CUL4B

## Abstract

In 2016 and 2018, Chung, Jansen and others described a new syndrome caused by haploinsufficiency of *PHIP* (pleckstrin homology domain interacting protein, OMIM *612,870) and mainly characterized by developmental delay (DD), learning difficulties/intellectual disability (ID), behavioral abnormalities, facial dysmorphism and obesity (CHUJANS, OMIM #617991). So far, *PHIP* alterations appear to be a rare cause of DD/ID. “Omics” technologies such as exome sequencing or array analyses have led to the identification of distinct types of alterations of *PHIP*, including, truncating variants, missense substitutions, splice variants and large deletions encompassing portions of the gene or the entire gene as well as adjacent genomic regions. We collected clinical and genetic data of 23 individuals with *PHIP*-associated Chung-Jansen syndrome (CHUJANS) from all over Europe. Follow-up investigations (e.g. Sanger sequencing, qPCR or Fluorescence-in-situ-Hybridization) and segregation analysis showed either *de novo* occurrence or inheritance from an also (mildly) affected parent. In accordance with previously described patients, almost all individuals reported here show developmental delay (22/23), learning disability or ID (22/23), behavioral abnormalities (20/23), weight problems (13/23) and characteristic craniofacial features (i.e. large ears/earlobes, prominent eyebrows, anteverted nares and long philtrum (23/23)). To further investigate the facial gestalt of individuals with CHUJANS, we performed facial analysis using the GestaltMatcher approach. By this, we could establish that *PHIP* patients are indistinguishable based on the type of *PHIP* alteration (e.g. missense, loss-of-function, splice site) but show a significant difference to the average face of healthy individuals as well as to individuals with Prader-Willi syndrome (PWS, OMIM #176270) or with a *CUL4B*-alteration (Intellectual developmental disorder, X-linked, syndromic, Cabezas type, OMIM #300354). Our findings expand the mutational and clinical spectrum of CHUJANS. We discuss the molecular and clinical features in comparison to the published individuals. The fact that some variants were inherited from a mildly affected parent further illustrates the variability of the associated phenotype and outlines the importance of a thorough clinical evaluation combined with genetic analyses for accurate diagnosis and counselling.

## Introduction

*PHIP* (pleckstrin homology domain interacting protein; OMIM *612870) was originally identified as a candidate gene for intellectual disability (ID) in one individual from a cohort of 100 ID-cases (de Ligt et al., 2012). Furthermore, microdeletions in the region 6q14.1, including *PHIP*, have been described in association with ID, developmental delay (DD) and dysmorphic features (Lespinasse et al., 2009; Van Esch et al., 2010; Becker et al., 2012). Webster et al. described in 2016 two individuals showing DD, ID, obesity and dysmorphisms, each having a *de novo* heterozygous predicted deleterious variant in *PHIP* (Webster et al., 2016). In 2018, Jansen et al. described 23 patients with different heterozygous mutations in *PHIP* and associated them with a syndrome mainly characterized by DD, learning difficulties/ID, behavioral abnormalities, facial dysmorphism and obesity (Jansen et al., 2018), later termed Chung-Jansen syndrome (CHUJANS, OMIM #617991) or DIDOD (Developmental delay, Intellectual Disability, Obesity, and Dysmorphism). Afterwards, Craddock et al. further characterized 10 individuals with predicted deleterious variants in *PHIP* and could show that the mutation spectrum is diverse and without any clustering or mutational hotspots (Craddock et al., 2019). Aoi et al. investigated a cohort of patients with suspected Cornelia-de-Lange syndrome (CdLS, OMIM #122470) and identified one individual with a missense substitution in *PHIP* (Aoi et al., 2019), while Kaur et al. extended the phenotypic spectrum with a case of CHUJANS also showing hypothyroidism and small kidneys (Kaur and Panigrahi, 2021). The latest publication reviewed 35 already reported individuals with *PHIP* variants together with one newly identified individual and sheds light on the impact of the variants on the structure of the protein by means of protein modeling (Dietrich et al., 2022).

*PHIP* encodes two protein isoforms, PHIP/DCAF14 (Pleckstrin Homology Domain Interacting Protein/DDB1- and CUL4-associated factor 14) and NDRP (Neuronal Differentiation-Related Protein). Both isoforms play a role in neurodevelopmental processes, such as E3 ubiquitination and neuronal differentiation (Jansen et al., 2018). Genotype/phenotype analyses suggest that *PHIP* haploinsufficiency is the cause of the described phenotypes of CHUJANS (Webster et al., 2016; Jansen et al., 2018; Craddock et al., 2019). Additionally, deficiency of *CUL4B*, a PHIP-interacting protein, has been previously associated with a very similar phenotype showing ID, central obesity, muscle wasting and dysmorphisms, due to disruption of the ubiquitin ligase pathway (syndromic X-linked intellectual developmental disorder; OMIM #300354) (Tarpey et al., 2007; Webster et al., 2016).

Altogether, 39 patients with variants in *PHIP* and five patients with deletions including *PHIP* have been published so far, and it still appears that *PHIP* variants are a rare cause of DD/ID.

In this study, we report 23 additional individuals with newly identified pathogenic or likely pathogenic *PHIP* variants or (partial) deletions of *PHIP.* Five of the described cases were proven to be inherited, either maternally or paternally, from a mildly affected parent. We performed facial phenotype analyses by the next-generation phenotyping approach GestaltMatcher. The phenotypic analysis was similarly extended to additional syndromes with molecular or clinical overlap with CHUJANS, namely *CUL4B*-related disorder and Prader-Willi syndrome (OMIM #176270).

Our findings further expand the mutational and phenotypic spectrum of *PHIP.* We discuss the molecular and clinical features in comparison to the already published individuals.

## Materials and methods

### Our cohort

Our cohort comprises 23 individuals of which thirteen were males, ten were females. They all presented in genetic departments due to DD/ID and/or behavioral changes. The data were collected thanks to a large international cooperation including Germany, Spain, Netherlands, Finland, France and Portugal. All individuals/legal guardians gave signed consent for anonymous publication of genetic and clinical data and 19 out of 23 individuals additionally consented to publication of photographs. Comprehensive clinical and genetic data were provided by the referring geneticists. Data were anonymized before sharing and ethics approvals were locally obtained.

### Genetic testing

Individuals had been analyzed by means of microarrays, gene panels or exome sequencing according to standard protocols at their respective institutions. Variants are mapped on the NM_017934.7 transcript.

### *PHIP* variants present in gnomAD

Known *PHIP* variants (ENST00000275034.4, corresponding to NM_017934) were retrieved from gnomAD v2.1.1 (Karczewski et al., 2020), limiting to loss-of-function, missense and synonymous single nucleotide variants (SNVs) or indels. The combined annotation-dependent depletion (CADD) score (Rentzsch et al., 2019) (https://cadd.gs.washington.edu/score) was calculated for each variant using GRCh37-v1.6 genomic coordinates. Amino acid positions of each protein region were retrieved from Uniprot (UniProt, 2021).

### GestaltMatcher analysis

GestaltMatcher (Hsieh et al., 2022) is a deep learning framework that quantifies the facial dysmorphic similarities among patients. GestaltMatcher was first trained on 3,438 frontal images with 139 different disorders from GestaltMatcher Database (https://db.gestaltmatcher.org/) to learn the facial dysmorphic features. It later encoded the photo into a 320-dimensional facial phenotype descriptor (FPD and spanned a clinical face phenotype space (CFPS)). The similarity between two patients can be quantified by the cosine distance in the CFPS.

We performed GestaltMatcher analyses on the following three cohorts: individuals with variants in *PHIP* (36 images), in *CUL4B* (27 images), and with Prader-Willi syndrome (PWS, 11 images). The *PHIP* cohort consisted of 18 frontal images from the unpublished cohort recruited in this study and 18 frontal images from previously published works - 14 images from Jansen et al. (2018), one image from Webster et al. (2016), one image from Aoi et al. (2019), and two images from Craddock et al. (2019). We collected *CUL4B* patients from four different publications, 13 images from Vulto-van Silfhout et al. (2015), seven images from Tarpey et al. (2007), four images from Cabezas et al. (2000), and three images from Badura-Stronka et al. (2010). We recruited 11 images of patients with PWS from the patient support group.

We first encoded each of the 74 images into FPDs and performed t-distributed stochastic neighbor embedding tSNE to visualize the distribution of patients in two-dimensional space.

### Average face analysis

We performed the average face analysis to visualize the different facial phenotypes in the three different cohorts (CHUJANS, *CUL4B*-related ID and PWS) and the healthy individuals. To simulate the cohort for the healthy individuals, we randomly selected ten healthy individuals that match the sex and age in UTKFace (Zhang et al., 2017) for each *PHIP* patient. Therefore, the healthy cohort contained 360 images. We then averaged the faces in the following four cohorts: *PHIP* (36 images), *CUL4B* (27 images), Prader-Willi syndrome (11 images), and healthy cohort (360 images) by first detecting the 68 facial landmarks and averaging over all the images in each cohort.

## Results

### *PHIP* variants

**FIGURE 1:**
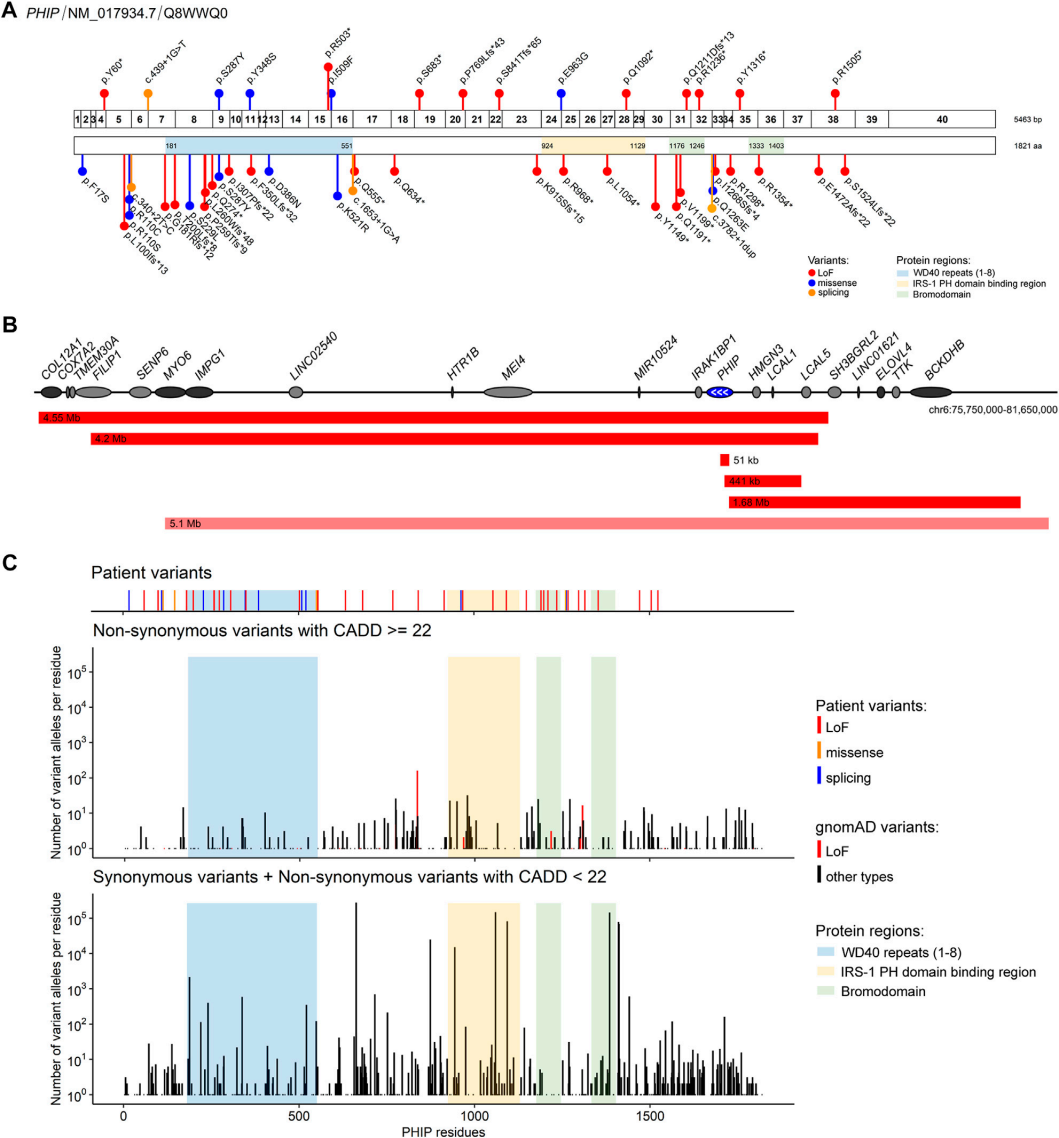
*PHIP* variants associated with Chung-Jansen syndrome identified in the present cohort and/or described in the literature **(A)** Schematic representation of *PHIP* exons (top; 1–40) and its encoded protein (bottom) showing the identified variants relative to the protein domains/regions. Variants identified in the present cohort are shown above, and the described in the literature are shown below. Variants are labelled with the nomenclature based on changes at protein levels except for splicing variants, for which coding DNA sequence nomenclature was used **(B)** Schematic representation of the deletions encompassing the *PHIP* gene (blue) identified in the present cohort (red) and in the literature (light red). Deletion sizes are shown. White arrows identify the direction of *PHIP* transcription. Genes in dark grey are, as *PHIP*, OMIM morbid genes **(C)** Comparison of the distribution of the *PHIP* variants identified in the present cohort and/or in the literature with the variants reported in gnomAD. Number of individuals with gnomAD variants were plotted for each combined codon. gnomAD variants were stratified by damage potential (based on variant type and CADD score).

The point variants described in our cohort are shown in Figure 1A. Fourteen variants in *PHIP* identified in our cohort had not been described before. One variant had previously been described in the literature (g.79735299G>T (hg19), c.860C>A, p.(Ser287Tyr)), (Craddock et al., 2019). Ten of the sequence variants were loss-of-function variants, four were missense variants and one affected a splice site. Three variants were found in two individuals each: one variant shared by two siblings, another found in a father and his daughter and another in two individuals that were not related to each other. The variants were classified according to ACMG criteria (Richards et al., 2015). The criteria used for classification as well as the variants can also be found in Table 1 and the Supplementary Table S1.

**TABLE 1:** Molecular and clinical features of *PHIP* individuals.

	Frequency Craddock et al., 2019 (n = 10)	Frequency (n = 25) Jansen et al., 2018, Webster et al., 2016	Frequency present cohort	1	2	3	4	5	6	7	8	9	10	11	12	13	14	15	16	17	18	19	20	21	22	23
Genotype				g.79735299G>T	g.79713575T>A	6q14.1 (79,719,926_79770881)x1 (51 kb)	6q14.1 (79770308–81454326)x1 (1.68 Mb)	g.79675705G>A	g.79671432_79671435del	g.79668268G>A	g.79664637dup	g.79787173_79787175del	6q14.1 (76086087_80285061)x1 (4.2 Mb)	g.79688310T>C	g.79724816G>A	g.79700597_79700600del	del (6) (q13q14.1) (75784986_80341335)x1 (4.55 Mb)	6q14.1 (79745807–80187057)x1 (441 kb)	g.79655835G>A	g.79655835G>A	g.79707284G>T	g.79707284G>T	g.79664637dup	g.79695088_79695089insTGTGTGTG	g.79770194C>A	g.79727252T>G
				c.860C>A	c.1525A>T			c.3274C>T	c.3631_3634del	c.3706C>T	c.3947dup	c.180_182del		c.2888A>G	c.1507C>T	c.2306_2309del			c.4513C>T	c.4513C>T	c.2048C>A	c.2048C>A	c.3947dup	c.2521_2522insCACACACA	c.439 + 1G>T	c.1043A>C
				p. (Ser287Tyr)	p. (Ile509Phe)			p. (Gln1092*)	p. (Gln1211Aspfs*13)	p. (Arg1236*)	p. (Tyr1316*)	p. (Tyr60*)		p. (Glu963Gly)	p. (Arg503*)	p. (Pro769Leufs*43)			p. (Arg1505*)	p. (Arg1505*)	p. (Ser683*)	p. (Ser683*)	p. (Tyr1316*)	p. (Ser841Thrfs*65)	p.?	p. (Tyr348Ser)
				CADD 27.6	CADD 32			CADD 41	CADD 33	CADD 39	CADD 33	CADD 33		CADD 35	CADD 39	CADD 32			CADD 39	CADD 39	CADD 39	CADD 39	CADD 33	CADD 33	CADD 35	CADD 26.5
gnomAD				-	-	-	-	-	-	-	-	-	-	-	1/250,618	-	-	-	-	-	-	-	-	-	-	-
Average age (yrs)	9	18	20	10	12	5	13	12	16	49	11	5	39	7	15	43	54	37	16	41	11	8	16	10	16	9
Sex	F:M = 5:5	F:M = 14:11	F:M = 10:13	M	M	F	F	M	F	M	M	F	F	F	F	F	M	M	F	M	M	M	M	M	M	F
Neonatal issues	Feeding difficulty (70%), Hypotonia (40%)	Feeding difficulty (33%)	Feeding difficulty (18%)	Icterus neonatorum	Plagiocephalus	Feeding difficulty	Feeding difficulty	Mild jaundice and neonatal hypoglycemia	-	Preterm issues	-	Feeding difficulty	Dehydratation and jaundice, feeding difficulty	Hip dysplasia, unilateral cong. Hip luxation	-	Suppletion for low iron	Clubfeet, hypotonia	Syndactyly of 2–3 toes	Feeding difficulty hypotonia	Hypotonia, exomphalos	Dyspnea	-	Newborn jaundice	Newborn jaundice	-	Cleft lip
DD	100%	100%	95.5%	+	+	+	+	+	+	+	+	+	+	+	+	+	+	+	+	+	+	+	-	+	+	+
ID	100%	92%	91.3%	+	-	+	+	+	+	+	+	+	+	-	+	+	+	+	+	+	+	+	+	+	+	+
Behavioral problems	100%	92%	86.96%	-	+	+	+	+	+	+	+	-	+	+	+	+	+	+	+	-	+	+	+	+	+	+
Facial Dysmorphism	100%	96%	100%	+	+	+	+	+	+	+	+	+	+	+	+	+	+	+	+	+	+	+	+	+	+	+
Other Dysmorphism	60%	79.2%	77.3%	+	+	+	-	+	+	+	+	+	+	+	+	-	+	+	+	+	+	-	+	+	+	+
Neurologic issues	Hypotonia (80%), Seizures (20%)	Hypotonia (32%)	Hypotonia (34.78%), Balance problems (30.43%), Seizures (17.39%)	+	+	-	+	+	+	+	+	+	+	+	+	+	+	+	-	-	-	-	+	+	+	+
Gastrointestinal problems	Constipation (70%)	Constipation (8%)	Constipation (34.78%)	-	-	-	+	+	-	-	-	+	-	+	+	+	+	-	-	-	+	-	-	-	-	-
Overweight/Obesity	30%	76%	69.6%	+	+	+	+	-	+	-	+	-	+	-	-	-	-	+	+	+	+	+	+	-	-	-

DD: developmental delay. ID: intellectual disability.

Five individuals showed *PHIP* haploinsufficiency due to a partial or complete deletion of *PHIP* as shown in Figure 1B. The size of the deletions varied from 51 kb to 4.55 Mb. Three of the deletions only partially affected *PHIP*: one intragenic deletion, another partially included *PHIP* in addition to three other genes (*HMGN3*, *LCAL1*, and *LCAL5*), and another partially included *PHIP* together with eight additional genes. The two remaining deletions encompassed the entire *PHIP* gene: one encompassed 16 genes, including *PHIP*, and the other included *PHIP* and 12 additional genes (one of which only partially). Although the deletion size and the number of genes involved vary among patients, the phenotypes are consistent and overlapping, hence suggesting that *PHIP* haploinsufficiency might be the major contributor to the disease of those patients.

Inheritance of the variants could be assessed in 15 individuals. Variants were found to be *de novo* in ten cases, whereas three variants were paternally inherited and two were maternally inherited. Due to unavailability of at least one of the parents, the origin of the variant could not be assessed in eight patients. In patients carrying a deletion affecting *PHIP*, one was proven to be *de novo*, one maternally inherited, one paternally inherited and in the two remaining cases inheritance was unknown.

In one individual (individual 1), whole exome analysis detected a second *de novo* variant in *CUX1* (NM_181552.4: c.3334del; p.(Gln1112Serfs*19)) which was classified as class 3 (unknown significance). The impact of the respective variant on the phenotype cannot be defined more precisely at present.

All variants of our cohort are either absent from gnomAD or present at a very low allele frequency. *PHIP* variants available in gnomAD were subsequently retrieved to verify whether specific domains of the PHIP protein might be depleted from putatively damaging variants. As shown in Figure 1C, less residues are affected by putatively damaging variants in gnomAD (CADD score ≥22) in comparison to putatively benign variants (synonymous variants and variants with CADD score <22).

### Most frequent clinical features

A summary of the main clinical features associated with *PHIP* alterations can be found in Table 1. Detailed clinical description of each patient of our cohort is available in Supplementary Table S1.

**FIGURE 2:**
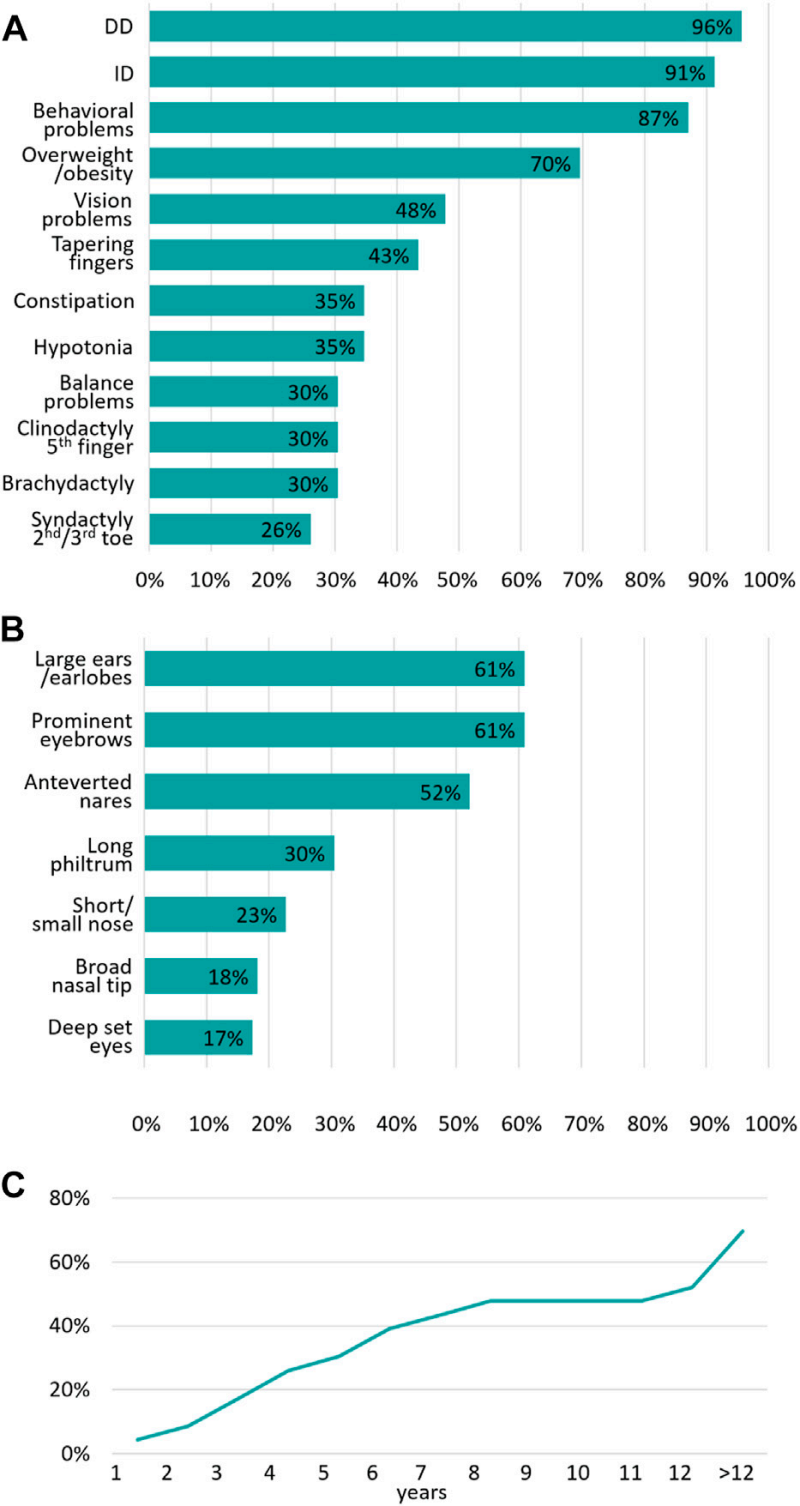
Summary of the main clinical features associated with *PHIP* variants **(A)** Summary of the most frequent clinical features of our cohort expressed as a percentage (n = 23) **(B)** Summary of the most frequent craniofacial dysmorphism of our cohort expressed as a percentage (n = 23) **(C)** Age of onset of overweight/obesity and the cumulative frequency expressed as a percentage.

In our cohort of 23 individuals with *PHIP* variants (SNVs, partial deletion, or deletion) the most common clinical features are DD (96%), followed by ID (91%) and behavioral problems (87%), as shown in Figure 2A and Table 1 as well as in the Supplementary Table S1. Overweight/obesity is the fourth most common feature (70%) in our cohort. Vision problems occurred in 48% of the individuals while tapering fingers were present in 43%. Constipation and muscular hypotonia were seen in 35%, followed by balance problems in 30%.

Interestingly, neonatal muscular hypotonia was only reported in 13% of the individuals, while it was more often reported later in life when the individuals were presented to a clinical geneticist (35%), which is important for the comparison with PWS patients.

### Craniofacial dysmorphisms

**FIGURE 3:**
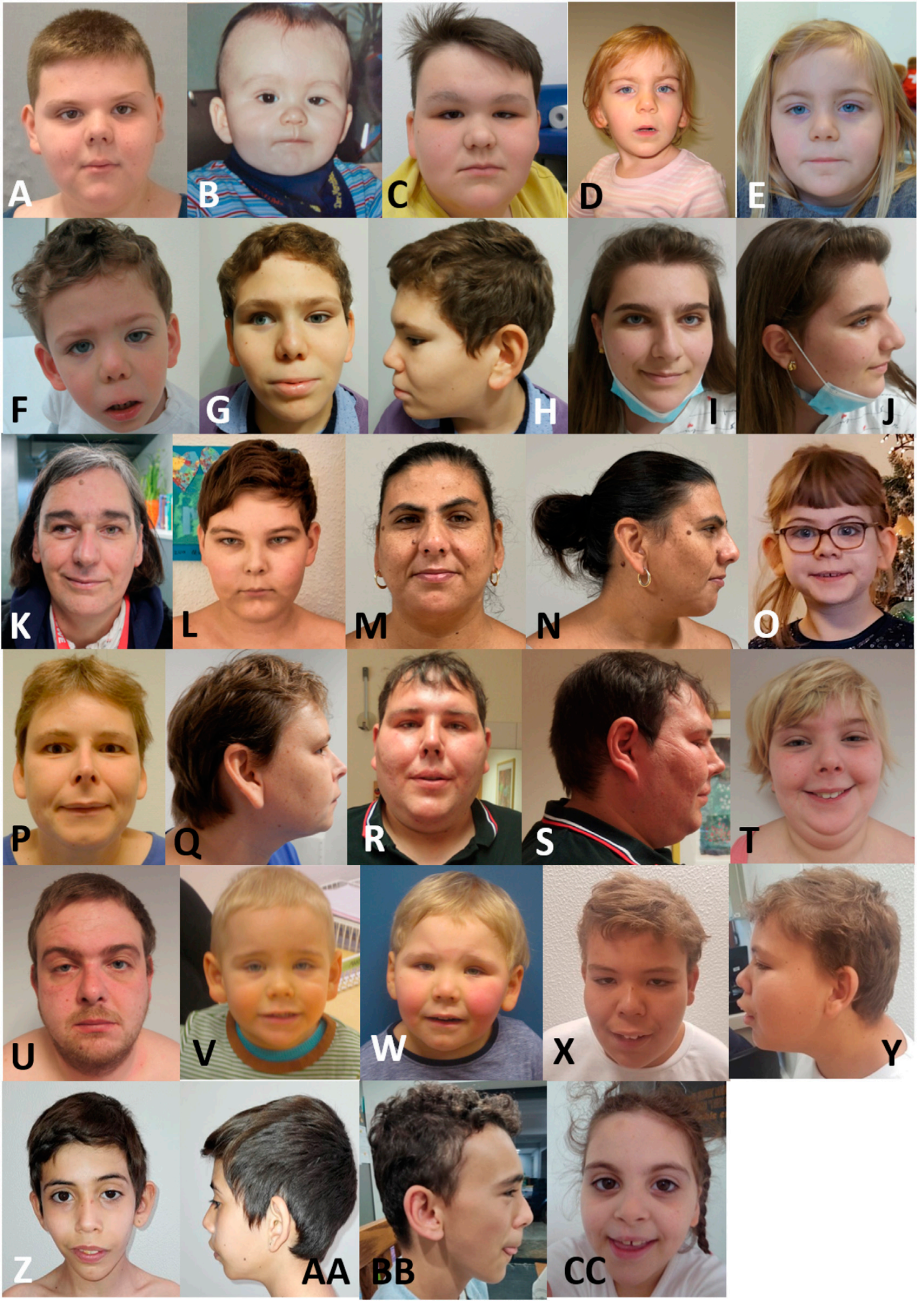
Facial appearance of the individuals of our cohort **(A)** Individual 1 (10 years) **(B,C)** individual 2 (1 year, 12 years) **(D,E)** individual 4 (3 years, 6 years) **(F–H)** individual 5 (3 years, 12 years, profile 12 years) **(I,J)** individual 6 (16 years) **(K)** individual 7 (49 years) **(L)** individual 8 (11 years) **(M,N)** individual 10 (39 years) **(O)** individual 11 (7 years) **(P,Q)** individual 13 (38 years) **(R,S)** individual 15 (37 years) **(T)** individual 16 (16 years) **(U)** individual 17 (41 years) **(V)** individual 18 (age unknown) **(W)** individual 19 (age unknown) **(X,Y)** individual 20 (16 years) **(Z,AA)** individual 21 (10 years) **(BB)** individual 22 (16 years) **(CC)** individual 23 (9 years).

All 23 individuals presented with characteristic craniofacial dysmorphic features (Figure 3). As shown in Figure 2B, the most common features are large ears/earlobes (61%) and prominent eyebrows (61%), followed by anteverted nares (52%). Less frequent dysmorphism comprise a long philtrum (30%), a short nose (23%), a broad nasal tip (17%) and deep-set eyes.

#### Limb dysmorphisms

**FIGURE 4:**
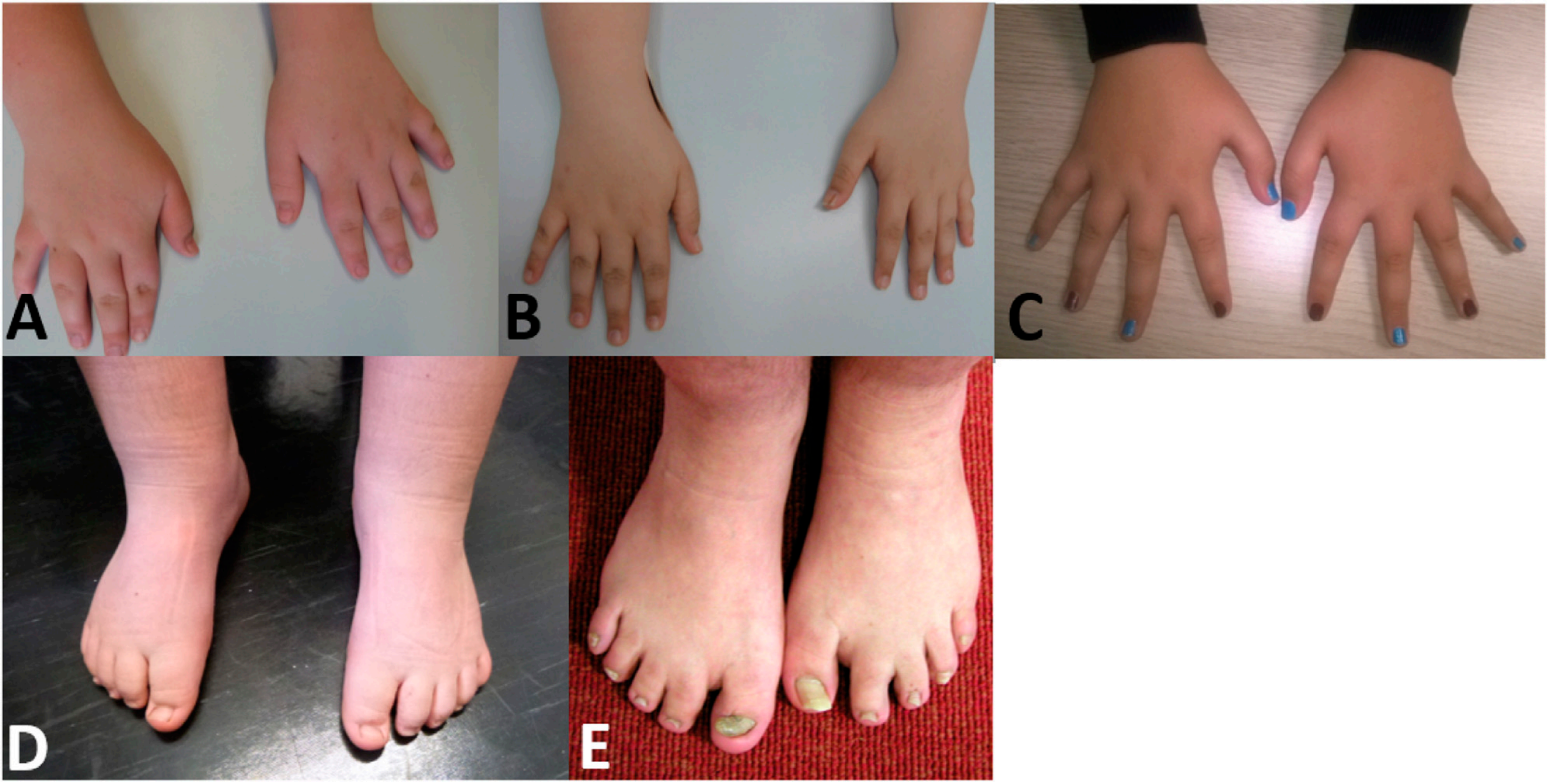
Pictures of individuals showing hand and/or feet anomalies **(A)** Hands of individual 1 (10 years) **(B)** hands of individual 2 (12 years) **(C)** hands of individual 6 (16 years) **(D)** feet of individual 1 (10 years) **(E)** feet of individual 17 (41 years).

Nearly half of the cohort presented tapering fingers (43%). 30% of the individuals showed brachydactyly, while clinodactyly of the fifth finger could be found in 30% of the individuals (Figure 4). 26% of the individuals showed syndactyly of the second/third toes.

### Age of onset of overweight/obesity

The age of onset of overweight/obesity appears to be highly variable. The incidence of this clinical feature increases as patients grow older (Figure 2C). In our cohort, obesity was present in 70% of the individuals. However, some of the individuals were examined at an age below 12 years, an age where obesity onset is not always observed. Based on our data, the incidence of overweight/obesity rises sharply during puberty.

30% of the individuals already show overweight/obesity at the age of 5 years. Almost 50% showed overweight/obesity at the age of 8 years, followed by a plateau phase with a second, sharp rise of incidence from the age of 12 years and above. The highest BMI in our cohort was 36.26 kg/m^2^ at the age of 15 years in a female individual.

### Facial gestalt analyzed by GestaltMatcher

**FIGURE 5:**
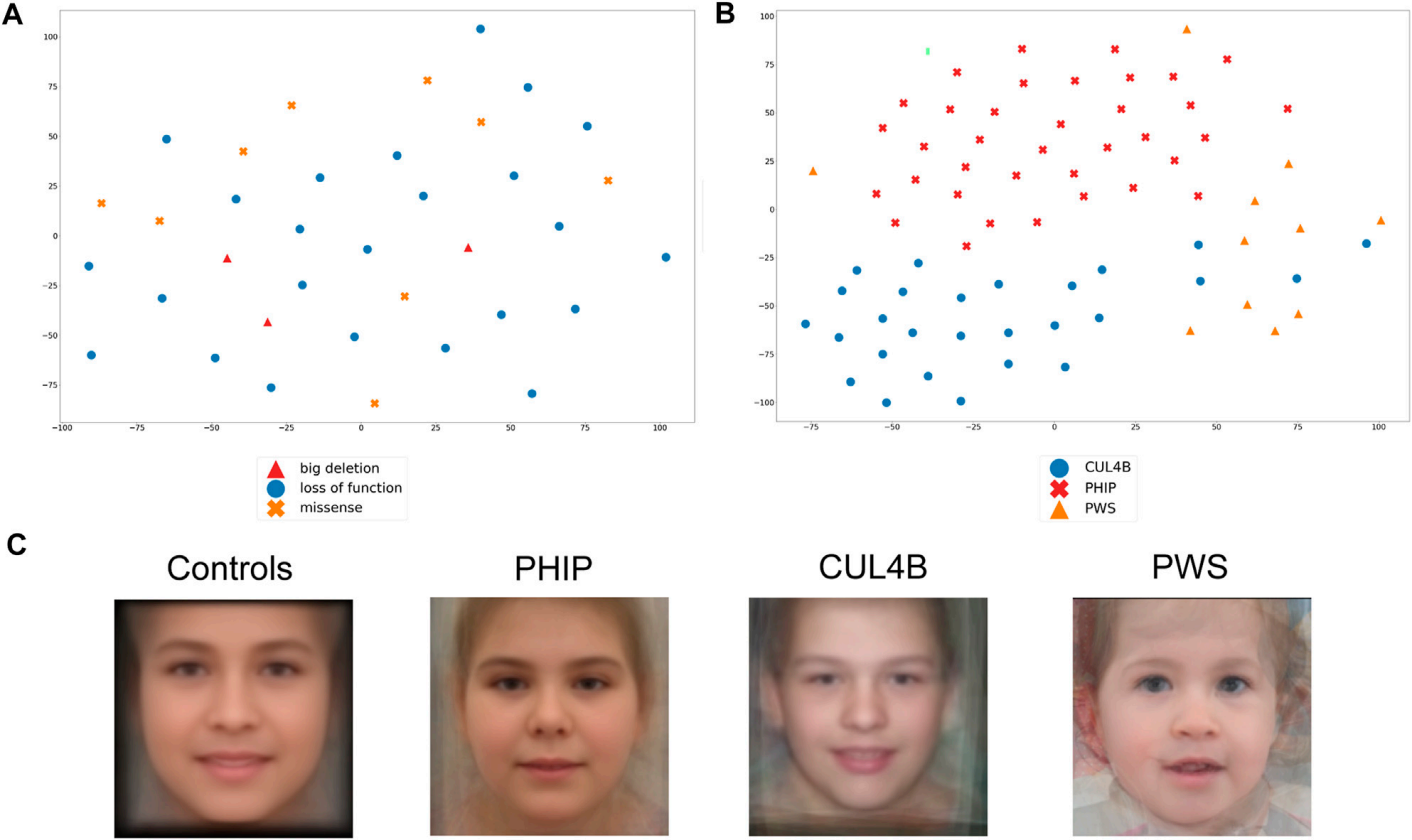
Analysis of the facial Gestalt with Gestaltmatcher. **(A)** tSNE analysis to validate whether *PHIP* individuals cluster based on the type of mutations (missense, loss-of-function, splice site). **(B)** tSNE analysis of patients with *PHIP* alteration, *CUL4B* alteration or PWS, which shows a clear phenotypic separation of the three conditions. **(C)** Average faces for healthy controls and each of the differential clinical diagnoses for CHUJANS, namely *CUL4B-*related disorders and PWS.

We subsequently took advantage of the GestaltMatcher approach to verify whether facial dysmorphisms are associated with a specific variant type and whether patients with different variants (large deletions, loss-of-function point variants, and missense substitutions) can be distinguished based on their facial features. As shown in Figure 5A, *PHIP* patients do not cluster based on variant type. Importantly, subtle differences might not be evident due to the small sample size.

We then extended the analysis and compared the facial gestalt of our patients with those of individuals with the clinical diagnosis of PWS as well as individuals with *CUL4B* variants*,* as both syndromes are characterized by ID and overweight and represent important differential clinical diagnoses (Figure 5B). PWS is one of the main clinical differential diagnoses that was suspected in almost all patients at a certain point. Furthermore, *CUL4B* and *PHIP* functionally interact on protein level as binding partners within the same protein complex. We could show that individuals with *PHIP* variants as well as individuals with *CUL4B* variants or PWS patients form distinct clusters, although some of *CUL4B* patients localize within the PWS cluster. The low image quality of some of the patients might explain why some patients behave as outliers.

We further generated average faces for individuals with *PHIP* SNVs or (partial) deletions, *CUL4B* variants and PWS, as well as a healthy control face. By this, we could see a difference in the facial gestalt from the *PHIP* cohort in comparison to the *CUL4B* and PWS cohort and from all the three cohorts to the average healthy control face, as depicted in Figure 5C.

## Discussion

Here we report on 23 new cases of *PHIP* associated CHUJANS. The shared clinical features include DD, ID, behavioral problems, overweight/obesity and some dysmorphic facial signs. Interestingly, the most common dysmorphic facial features are large ears/earlobes, prominent eyebrows and anteverted nares. This is in line with the previously reported cohorts of patients (Wentzel et al., 2010; Becker et al., 2012; Webster et al., 2016; Jansen et al., 2018; Craddock et al., 2019).

### Developmental delay and ID

Overall, 96% of the individuals of our cohort showed some kind of DD. In some cases, either motor or speech development was affected. One case presented with normal development until the age of 3 years, followed by a delay of psychomotor development after this age. This reveals that developmental milestones up to the first years of life are not always disrupted, contrary to previous findings (Craddock et al., 2019). ID was observed in 91% of the individuals in our cohort, but only three of our patients presented with an IQ below 50. This is in agreement with the findings of Craddock and others, who reported that intellectual disability is generally mild and characterized by a better verbal performance than IQ (Craddock et al., 2019). Accordingly, five parents of our cohort were diagnosed as mildly affected only after the identification of the genetic cause of the disease of their respective children. In the previously published cohorts, only two inherited cases were described (Jansen et al., 2018).

### Overweight/obesity

In our study, we found a different time of onset of overweight or obesity. Whereas some patients started gaining weight during childhood, others started being overweight only during puberty. Overall, nearly 70% of the individuals of our cohort showed overweight or obesity. Since obesity was defined as one of the main criteria of the also called “DIDOD-Syndrome” (developmental delay, intellectual disability, OBESITY and dysmorphism), one should be careful not to miss the diagnosis of CHUJANS as patients might not show obesity or overweight at a younger age. This should be considered for phenotype-based filtering processes of exome or genome data.

Marenne et al. (2020) were able to demonstrate that mutations in *PHIP* are causing overweight through PHIP interaction with Proopiomelanocortin (POMC). They could establish that loss-of-function variants in *PHIP* are more frequently associated with obesity or overweight than missense substitutions. Our study cannot confirm their finding that missense variants in *PHIP* do not lead to obesity, as two out of four individuals with a missense variant in our cohort showed obesity already during childhood.

In addition, Marenne and others also identified patients with *PHIP* variants and obesity but without developmental delay (Marenne et al., 2020). They concluded that *PHIP* variants might affect transcriptional regulation and different types of mutations result in variable clinical outcomes. They suggested that *PHIP* should be included in genetic testing recommended in clinical guidelines as part of the assessment for severe childhood-onset obesity (Marenne et al., 2020).

Our findings confirm the importance of *PHIP* variants in the context of obesity onset. However, as the age range of onset of obesity in all our obese patients was rather wide, from early childhood to puberty (>12 years), *PHIP* haploinsufficiency should be taken into account also in the absence of overweight/obesity. We also suggest following up on the overweight/obesity as longitudinal data are missing for this relatively newly defined syndrome (Chung-Jansen syndrome or DIDOD syndrome, OMIM #617991) (de Ligt et al., 2012; Webster et al., 2016; Jansen et al., 2018; Craddock et al., 2019). It would be interesting to assess if all individuals with a reduced dosage of functional *PHIP* gene product show overweight/obesity in later adult years.

As PWS is also a DD/ID/obesity syndrome, it is a common clinical differential diagnosis to CHUJANS. When PWS testing is negative, CHUJANS should be considered. Thus, *PHIP* should also be included in obesity panels.

### Behavioral problems

Behavioral problems have been described as one of the most common features of patients with *PHIP* variants. Our data highlight this relevance since 87% showed behavioral problems which were not necessarily present at younger ages. The types of behavioral changes are very broad and include loss of self-control and impulsiveness, aggressions, anxiety, autism spectrum disorder and motor hyperactivity. A large variety of behavioral problems have also been described in other patients (Jansen et al., 2018; Craddock et al., 2019). Interestingly, longitudinal follow-up on one patient revealed that behavioral problems worsened with increasing age. By the late teenage years, the patient had already been in the psychiatric ward several times. The worsening of his behavioral disturbances made it impossible for this patient to live at home with his family. We suggest a regular follow-up on the behavioral problems of patients diagnosed with *PHIP* in childhood, adolescence and adulthood to learn more about this main feature of the syndrome.

### Facial gestalt

The main craniofacial dysmorphisms are large ears/earlobes and prominent eyebrows, followed by anteverted nares and, much less often, by a long philtrum, a short nose, a broad nasal tip and deep-set eyes. This is in line with previous reports (Webster et al., 2016; Jansen et al., 2018; Craddock et al., 2019).

In our cohort, we could not show any clustering of the facial phenotype depending on the type of genetic variant or deletion affecting *PHIP*. This may also be due to the small cohort size.

Van der Donk and others had previously shown that patients with a pathogenic variant in *PHIP* have a characteristic facial gestalt (van der Donk et al., 2019). Because of possible overlapping facial features, we compared the facial gestalt of individuals with variants affecting *PHIP* with the facial gestalt of published individuals with *CUL4B* alterations. Since PHIP/DCAF14 is one of the multiple substrate receptors of the proteolytic CUL4-DDB1 (Jansen et al., 2018; Tirado-Class et al., 2022), we postulated a phenotypic overlap between patients with *PHIP* or *CUL4B* variants. Furthermore, both proteins are involved in fork stability and genome integrity (Tirado-Class et al., 2022). GestaltMatcher did not confirm this hypothesis and rather showed that each disorder shows its distinct characteristic facial phenotype. We also compared the facial phenotype to that of the clinically relevant differential diagnosis PWS (which was suspected in almost all patients of our cohort at a certain time point). GestaltMatcher confirmed the hypothesis that the facial gestalt of both conditions differs. The low image quality of some of the patients might explain why some patients behave as outliers.

In summary, we conclude that the facial gestalt of individuals with *PHIP* alterations leading to CHUJANS is quite specific and recognizable and can be identified by using next-generation phenotyping approaches like GestaltMatcher.

### Comparison of phenotypes: CHUJANS vs. Cabezas syndrome and PWS

Since the two proteins PHIP and CUL4B belong to the same complex and are involved in the same processes (DNA repair, fork stability and genome integrity), as mentioned before, we chose individuals with *CUL4B* associated Cabezas syndrome to investigate whether their facial gestalt overlaps with that of CHUJANS or can be clearly distinguished by next-generation phenotyping approaches like GestaltMatcher (reported in 4.4). To compare the clinical phenotype of CHUJANS with that of Cabezas syndrome and that of PWS beyond facial features, we contrasted the clinical features of our cohort with those of published cases (2000–2019) of Cabezas syndrome, summarized by López et al., 2020, and the clinical features typical of PWS according to GeneReviews^®^ (see Supplementary Table S2). This revealed that ID is the most common clinical feature of these syndromes while DD and speech delay are also found in nearly all individuals affected by Cabezas syndrome and PWS but only in about half of the individuals having CHUJANS. Other frequent findings in Cabezas syndrome seem to be short stature, sandal gap and short feet (Lopez et al., 2020) which are very rarely found in our cohort of CHUJANS. Development of short stature and obesity, key features in PWS, is favorably influenced by the growth hormone therapy that is part of PWS clinical management. Obesity, however, was described in about half of the affected individuals in Cabezas syndrome while nearly 70% of individuals with CHUJANS became overweight/obese. Tremor is common in Cabezas syndrome, which has not been reported in our cohort and in PWS. Nevertheless, balance problems have occurred in 7/23 individuals of our CHUJANS cohort. Behavioral changes are a little less frequent in Cabezas syndrome than in our CHUJANS cohort. Tarpey et al. reported that 12 out of 15 individuals showing Cabezas syndrome have aggressive outbursts (Tarpey et al., 2007). This feature has also been reported in our cohort but only in 6/23 individuals. Overall, 86.4% of our cohort show a kind of behavioral change, but aggressions seem not to be the most common one. An age dependent, characteristic behavior profile is observed in almost all individuals with PWS. In summary, not only facial gestalt but also clinical characteristics of CHUJANS differ from those of Cabezas syndrome and of PWS.

## Conclusion

The phenotype of *PHIP*-associated disorders is variable, although some common features can be recognized. Even though it is still a rare finding, we suggest including *PHIP* in diagnostic gene panels specific for developmental delay, ID, behavioral abnormalities and/or obesity. This could help find the genetic cause for unsolved cases of DD/ID and/or behavioral problems as well as obesity in childhood and adulthood. In turn, the discovery of new patients with *PHIP* variants would elucidate further the variability of the associated clinical phenotype. In addition, we suggest taking advantage of facial gestalt software such as GestaltMatcher to assist with the clinical classification of patients.

## Data Availability

The original contributions presented in the study are included in the article/Supplementary Material, further inquiries can be directed to the corresponding authors.
